# Prevalence of Postpartum Family Planning Service Coverage in Selected Referral Facilities of Nepal

**DOI:** 10.31729/jnma.4788

**Published:** 2020-01-31

**Authors:** Kusum Thapa, Rolina Dhitai, Sameena Rajbhandari, Shikha Thapa, Sabina Pokhrei, Sangeeta Mishra, Shanti Subedi, Dela Singh, Shreedhar Acharya, Sunil Mani Pokhrei, Kalpana Thapa, Atit Poudel, Sapana Vaidya, Emily Anne Tunnacliffe, Anita Makins, Sabaratnam Arulkumaran

**Affiliations:** 1 Nepal Society of Obstetricians and Gynaecologist, Paropakar Maternity and Women's Hospital, Thapathali, Kathmandu, Nepal; 2 Koshi Zonal Hospital, Biratnagar, Morang, Nepal; 3 Nobel Medical College Teaching Hospital, Biratnagar, Morang, Nepal; 4 Western Regional Hospital Pokhara, Nepal; 5 Lumbini Zonal Hospital, Butwal, Nepal; 6 Bharatpur Hospital, Bharatpur, Nepal; 7 Bheri Zonal Hospital, Nepalgunj, Nepal; 8 Bhakatpur Hospital, Bhaktapur, Nepal; 9 international Federation of Obstetrics and Gynecology, London, United Kingdom

**Keywords:** *family planning services*, *female sterilization*, *intrauterine devices*, *postpartum period*

## Abstract

**Introduction:**

Nepal Society of Obstetricians and Gynecologists jointly with the Nepalese government and with the support from the International Federation of Obstetrics and Gynecology has implemented an initiative to institutionalize postpartum family planning services in selected major referral facilities of Nepal to address the gap of low uptake of postpartum family planning in Nepal. The aim of the study is to find the prevalence of the service coverage of postpartum contraception in the selected facilities.

**Methods:**

A descriptive cross-sectional study was conducted in seven major referral facilities across Nepal. Data were collected from the hospital records of all women who delivered in these facilities between October 2018 and March 2019. Ethical approval for this study was obtained from Nepal Health Research Council. Data analysis was done with SPSS version 23.

**Results:**

Among the 29,072 deliveries from all the facilities, postpartum family planning counseling coverage was 27,301 (93.9%). The prevalence of uptake of Postpartum Intrauterine Device is 1581 (5.4%) and female sterilization is 1830 (6.3%). In total 11387 mothers (52.2%) had the intention to choose a postpartum family planning method. However, 36% of mothers neither used nor had the intention to choose a postpartum family planning method.

**Conclusions:**

The coverage of Postpartum Intrauterine Device counseling service coverage in Nepal is higher in 2018 as compared to 2016-2017 and in other countries implementing Postpartum Intrauterine Device initiatives. However, the prevalence of service coverage of immediate Postpartum Family Planning methods, mainly Postpartum Intrauterine Device in 2018 is lower in Nepal as compared to 2016-2017, and other countries implementing Postpartum Intrauterine Device initiative. More efforts are needed to encourage mothers delivering in the facilities to use the postpartum family planning method.

## INTRODUCTION

Nepal Society of Obstetricians and Gynecologists (NESOG) jointly with the Nepalese government and with the support from International Federation of Obstetrics and Gynecology (FIGO) had implemented the initiative of institutionalizing immediate postpartum family planning (PPFP) services in selected major referral facilities of Nepal between 2015 and 2019.^1–3^ The initiative had focused on PPFP services that could be incorporated as a routine part of maternity care in the selected hospitals. The outcome of institutionalization was to improve the coverage of PPFP counseling and uptake of immediate PPFP methods.^1–3^

The increasing institutional deliveries in Nepal provides a one-stop approach to provide maternity and PPFP service at the same time.^4^ However, the data on postpartum family planning service coverage from the health facilities in Nepal remains limited.

This study aims to assess the service coverage related to postpartum contraception in selected referral health facilities in Nepal that were implementing PPFP programs.

## METHODS

We conducted this descriptive cross-sectional study in seven referral facilities implementing the PPFP initiative across Nepal. The data was collected from Koshi Zonal Hospital (KZH), Lumbini Zonal Hospital (LZH), Western Regional Hospital (WRH), Bheri Zonal Hospital (BZH), Bharatpur Hospital (BH), Bhaktapur Hospital (BKTH), and Nobel Medical College Teaching Hospital (NMCTH) for a period of 6 months between October 2018 and March 2019. We obtained ethical approval for this study from the Nepal Health Research Council. Permission to conduct the study was also obtained from all the seven health facilities. We collected the anonymized data from all the facility records.

The maternity registers had details of deliveries as well on the uptake of immediate PPFP methods which included female sterilization and Postpartum Intrauterine Device (PPIUD). Additionally, as part of the study, a separate register was used by the implementing sites to assess the coverage of counseling on PPFP in each facility. All the registered delivery in each of the 7 hospitals were included in the study. Data with insufficient variables, improperly registered data, and unreadable data were excluded from the study.

As a descriptive study using hospital records, the aim of this study was to assess the overall service coverage. As the data of all the deliveries recorded within the study period is included in this study, we did not perform inferential statistical analysis and performed descriptive analysis only for interpretation.^5^

Nevertheless, the minimum required sample size was calculated to be 28,782, using the formula for a finite population^6^ based on the annual delivery across all the 7 facilities as follows.

n = N*X / (X + N - 1)

where,

X = Z_α/2_^2^ *p*(1-p) / MOE^2^,

and Z_α/2_ is the critical value of the Normal distribution at α/2 for a confidence level of 95%, α is 0.05 and the critical value is 1.96, MOE is the margin of error at 0.40%, p is the sample proportion at 36% based on the prevalence from previous study^4^ and N is the population size of 60,000 based on the annual deliveries across the 7 facilities.

Considering incomplete data, we increased the sample size to 29,072 records of all deliveries between October 2018 and March 2019. The data is presented in numbers and percentages. Data analysis was done using SPSS version 23.

## RESULTS

The counseling coverage was 27,301 (93.9%) of all the 29,072 deliveries across all seven sites. NMCTH and BKTH which were the newest implementing facilities had 4,084 (99.6%) and 625 (100%) PPFP counseling coverage (Table 1).

**Table 1:** Counseling coverage of PPFP for mothers in the selected facilities.

Hospitals	Deliveries during data collection	Counseling coverage n (%)
KZH	4879	4410 (90.4)
BZH	2643	2601 (98.4)
LZH	4945	4531 (91.6)
WRH	4510	4014 (89.0)
BH	7371	7036 (95.5)
BKTH	625	625 (100)
NMCTH	4099	4084 (99.6)
Total counseling coverage	29072	27301 (93.9)

Among all the mothers who gave birth in the selected facilities, 3411 (11.7%) used a PPFP method immediately after childbirth. In total, 1581 mothers chose to use PPIUD from selected seven facilities accounting for 5.4% of all the deliveries. Among the mother who gave birth in each facility, the proportion of uptake of PPIUD was the highest in BKTH 625 (26%) and the lowest in LZH 4945 (2%) deliveries. In total, 1830 mothers from the seven facilities chose to have female sterilization immediately after childbirth accounting for 6.3% of all the deliveries. The proportion of mothers undergoing female sterilization was observed to be the highest in the two facilities in Province One with 706 (17%) in NMCTH and 679 (14%) in KZH. The overall proportion of women using an immediate PPFP method including both PPIUD and female sterilization was highest in BKTH and lowest in BH (Table 2).

**Table 2:** Immediate PPFP uptake among mothers in the selected facilities.

Hospitals	Deliveries during data collection	PPIUD uptake	Female sterilization	Overall uptake of immediate PPFP method
		n (%)	n (%)	n (%)
KZH	4879	345 (7.1)	679 (13.9)	1024 (21.0)
BZH	2643	267 (10.1)	34 (1.3)	301 (11.4)
LZH	4945	96 (1.9)	137 (2.7)	233 (4.6)
WRH	4510	168 (3.7)	177 (3.9)	345 (7.6)
BH	7371	246 (3.3)	80 (1.1)	326 (4.4)
BKTH	625	148 (23.7)	17 (2.7)	165 (26.4)
NMCTH	4099	311 (7.6)	706 (17.2)	1017 (24.8)
Total uptake	29072	1581 (5.4)	1830 (6.3)	3411 (11.7)

The other PPFP methods include the options that are provided to women after 6 weeks of childbirth in Nepal. The combined oral contraceptive pills containing estrogen are provided only 6 months after childbirth in Nepal. The details of the methods and the timing of using these different PPFP methods are provided to the mothers during PPFP counseling in the selected facilities. Among mothers who preferred other PPFP methods, the highest proportion was observed for injectables accounting for 4102 (16.4%) of the total deliveries in the seven facilities, followed by 669 (14.4%) women prefer their husbands to have male sterilization. In total, 1887 (7.1%) of mothers giving birth in the seven facilities preferred implants followed 1779 (4.9%) women preferring male condoms, and 1073 women preferring interval IUD (4.1%). The lowest preferences were for oral contraceptive pills by only 897 (2.6%) women and natural methods such as withdrawal methods by 980 (2.4%) women (Table 3).

**Table 3:** Intention to choose different methods of PPFP available in the selected facilities by the mothers in the later part of their postpartum period.

Hospitals	Male sterilization	Male condom	Interval IUCD	Implant	Injectable	Oral contraceptives	Withdrawal	Overall Intention
	n (%)	n (%)	n (%)	n (%)	n (%)	n (%)	n (%)	
KZH	15 (0.3)	106 (2.2)	316 (6.5)	434 (8.9)	858 (17.6)	144 (2.9)	11 (0.2)	1884 (38.6)
BZH	59 (2.2)	112 (4.2)	146 (5.5)	219 (8.3)	555 (20.9)	86 (3.2)	1 (0.04)	1178 (44.3)
LZH	76 (1.5)	87 (1.8)	123 (2.5)	120 (2.4)	156 (3.1)	42 (1)	2 (0.04)	606 (12.3)
WRH	96 (2.1)	44 (0.9)	35 (0.7)	73 (1.6)	101 (2.2)	24 (0.5)	13 (0.4)	386 (8.4)
BH	402 (5.7)	1214< (17.2)	300 (4.1)	579 (7.8)	999 (13.6)	494 (6.7)	938 (12.7)	4926 (67.8)
BKTH	18 (2.9)	23 (3.7)	44 (7.0)	67 (10.7)	163 (26.1)	4 (0.6)	12 (1.9)	331 (52.9)
NMCTH	3 (0.1)	193 (4.7)	109 (2.7)	395 (9.6)	1270 (31.1)	103 (2.5)	140 (3.4)	2213 (54.1)
Total intention	669 (14.4)	1779 (4.9)	1073 (4.1)	1887 (7.1)	4102 (16.4)	897 (2.6)	980 (2.7)	11387 (52.2)

## DISCUSSION

This study shows that the institutionalization process of PPFP services has resulted in high coverage of more than 90% PPFP counseling services of the deliveries across all the implementing facilities. However, the uptake of immediate PPFP methods such as PPIUD and female sterilization remained low at 12% of the total deliveries. Over 50% of the mothers intended to use a PPFP method at some point. However, 36% of the mothers neither took up any PPFP method nor had the intention to use any PPFP method later.

In this study, though the counseling coverage seemed consistent across all the facilities, there was a variation in the uptake of immediate PPFP methods. Bhaktapur hospital which had started the initiative more recently had the highest service coverage for PPIUD at 24% of all the deliveries.Studies suggest that reduced ratios of patients to health providers improve the quality of care and patient satisfaction.^7–9^

This study showed that over 50% of women giving childbirth across seven facilities intended to use a PPFP method at some point. Injectable was the most commonly intended method which is consistent with the national data in Nepal.^10^ The second most common intended method among the postpartum mothers was male sterilization of their partners. PPFP programs focus primarily on the methods used by women only and the methods used by their male partners are not given sufficient attention. The implant was the third commonly preferred PPFP method among mothers. The World Health Organization guidelines suggest that implant can be used by the mothers immediately after childbirth.^11^ Although it has been discussed, the implant has not yet been introduced as an immediate PPFP method in Nepal.

A majority of the mothers have the intention to choose a PPFP method, however, the services are not readily available in the immediate postpartum period. Methods such as implants, female sterilization after vaginal delivery and male sterilization are approaches that could be provided in the immediate postpartum period to mothers and their male partners. However, these methods are not considered as options for immediate PPFP, limiting method choice and subsequent uptake of contraception. In order to meet the 2020 FP goals,^12^ the supply must meet the demand, and so we must strive to make these methods an integral part of immediate PPFP services.

This study has certain limitations. First, the length of the study is too short due to the time limitation of the initiative. A longer study would have provided an opportunity to carefully follow up the trend in the long run. Second, this study only provides descriptive data on the selected facilities and does not provide inferential statistics. Detailed characteristics of the mothers from the facilities could have enriched the results and interpretation of this study and plan for the future programs at larger scales. However, the qualitative study from previous phases had already reflected the mothers' perception regarding PPFP from other facilities.^3^

## CONCLUSIONS

This study reflects the findings of a PPFP program in Nepal. The study showed that improving PPFP counseling coverage alone may not necessarily improve the uptake of PPFP methods. Moreover, the intention of mothers to use different PPFP methods highlights the need to find a balance between demand and supply which addresses the unmet need of PPFP. The findings also highlight that PPFP programs in Nepal must assess factors influencing service uptake and be flexible in its approaches in order that the program can be continuously adapted according to lessons learned.
